# Aspirin Use and the Incidence of Hepatocellular Carcinoma in Patients With Hepatitis B Virus or Hepatitis C Virus Infection: A Meta-Analysis of Cohort Studies

**DOI:** 10.3389/fmed.2020.569759

**Published:** 2021-01-08

**Authors:** Xiaofei Li, Shuang Wu, Yuexiao Yu

**Affiliations:** Department of Infectious Diseases, Yiwu Central Hospital, Yiwu, China

**Keywords:** aspirin, hepatocellular carcinoma, hepatitis B virus, hepatitis C virus, cirrhosis, gastrointestinal bleeding, meta-analysis

## Abstract

**Background:** The association between aspirin use and the incidence of hepatocellular carcinoma (HCC) in patients with hepatitis B virus (HBV) or hepatitis C (HCV) virus infection remains not fully determined. A meta-analysis was performed to summarize the findings of cohort studies.

**Methods:** Relevant cohort studies were retrieved via a search of PubMed Cochrane's Library and Embase databases. A random-effect model was used to pool the results. Subgroup analyses were performed to evaluate the influence of study characteristics on the association.

**Results:** Seven cohort studies with 120,945 adult patients with HBV or HCV infection were included. Pooled results showed that aspirin use was independently associated with a reduced risk of HCC in these patients (risk ratio: 0.73, 95% confidence interval: 0.64 to 0.83, *p* < 0.001; I^2^ = 86%). Subgroup analyses showed that aspirin use was associated with a reduced HCC risk regardless of the viral type, age, sex, the diabetic, and cirrhotic status of the patients, and the follow-up durations. Moreover, consistent results were obtained in studies with and without adjustment of antiviral treatment and statin use. Pooled results of four studies showed that aspirin use was associated with an increased risk of gastrointestinal bleeding in these patients (risk ratio: 1.15, 95% confidence interval: 1.02 to 1.28, *p* = 0.02; I^2^ = 0%).

**Conclusions:** Aspirin use was independently associated with a reduced risk of HCC in patients with HBV or HCV infection, whereas the risk of gastrointestinal bleeding may be increased. These results should be validated in clinical trials.

## Introduction

Hepatocellular carcinoma (HCC) is a common malignancy of the digestive system (1). Epidemiology studies showed that each year, ~500,000 patients were diagnosed with HCC worldwide (1). Moreover, treatment strategies for HCC remain limited in current clinical practice, which attributes to the relatively high mortality in these patients (2, 3). Hepatitis B virus (HBV) or hepatitis C virus (HCV) infection is the known risk factor for the development of HCC (4–6). Although extensive use of medications for HBV suppression or HCV eradication could reduce the incidence of HCC in these patients, patients with HBV or HCV infection remain at high risk for the development of HCC (7, 8). Accordingly, prophylactic measures for HCC in these high-risk patients remain of great clinical significance (9).

Aspirin (acetylsalicylic acid) is a commonly used nonsteroidal anti-inflammatory drugs (10). Via irreversible inactivation of cyclooxygenase, aspirin exerts multiple pharmacological effects, including antiplatelet and anti-inflammation (11). Aspirin is a cornerstone for the prevention and treatment of atherosclerotic cardiovascular diseases (12). Moreover, increasing evidence suggests that aspirin may have anticancer efficacy (13). The use of aspirin has been related to reduced incidences of endometrial cancer (14), gastric cancer (15), digestive tract cancers (16), pancreatic cancer, among others (17), whereas studies that evaluated the association between aspirin use and risk of HCC in patients with HBV or HCV infection showed inconsistent results (18–24). Although a recent meta-analysis showed that aspirin might be associated with a reduced risk of HCC in a mixed population, meta-analyses limited to patients with HBV or HCV infection have not been performed (25). Besides, some large-scale cohort studies (22–24) focusing on the association between aspirin use and HCC risk in patients with HBV or HCV infection have been published but never been evaluated in a meta-analysis. Moreover, whether aspirin use is associated with an increased risk of gastrointestinal bleeding (GIB) in patients with HBV or HCV infection remains unknown. Therefore, in this study, we aimed to perform a meta-analysis of cohort studies to systematically evaluate the influence of aspirin use on the risks of HCC and GIB in patients with HBV or HCV infection.

## Methods

The meta-analysis was performed in accordance with the Meta-analysis of Observational Studies in Epidemiology (26) and Cochrane's Handbook (27) guidelines.

### Literature Search

Studies were identified via a systematic search of electronic databases of PubMed, Cochrane's Library, and Embase via the following combined terms: (“aspirin” OR “antiplatelet”) AND (“chronic hepatitis B” OR “chronic hepatitis C” OR “hepatitis B virus” OR “hepatitis C virus” OR “HBV” OR “HCV”). The combined terms were entered into the databases as a single search. We used this keywords search strategy instead of those searched as “text words” or as “Mesh terms” to retrieve more comprehensive records. The search was limited to human studies without language restriction. The reference lists of related original and review articles were also analyzed using a manual approach. The final literature search was performed on November 19, 2020.

### Study Selection

The inclusion criteria for the studies were (1) cohort studies published as full-length articles; (2) included at least 1,000 adult patients with HBV or HCV infection but without HCC at baseline; (3) evaluated the association between aspirin use and the incidences of HCC (primary outcome) and/or GIB (secondary outcome) during a follow-up duration >1 year; and (4) reported the relative risk for the association with multivariate analyses and adjustment of confounding factors. Reviews, editorials, preclinical studies, and studies irrelevant to the aim of the current meta-analysis were excluded. Conference proceedings and unpublished data were not considered for this meta-analysis because these reports may not be strictly peer-reviewed, and including of these reports may confound the results of the meta-analysis.

### Data Extracting and Quality Evaluation

Literature search, data extraction, and quality assessment of the included studies were performed according to the predefined inclusion criteria independently by two authors. Discrepancies were resolved by consensus or discussion with the corresponding author. The extracted data included (1) name of the first author, publication year, and country where the study was performed; (2) study design characteristics; (3) patient characteristics, including disease status, sample size, age, sex, prevalence of diabetes, and proportions of patients with cirrhosis at baseline; (4) dose and definition of aspirin use; (5) follow-up durations; (6) strategy for HCC validation and number of HCC cases during follow-up; and (7) confounding factors that were adjusted in the multivariate analyses. The quality of each study was evaluated using the Newcastle–Ottawa Scale (28), which ranges from 1 to 9 stars and judges each study regarding three aspects: selection of the study groups; the comparability of the groups; and the ascertainment of the outcome of interest.

### Statistical Analyses

Risk ratios (RRs) and their corresponding 95% confidence intervals (CIs) were selected as the general measure for the association between aspirin use and HCC risk in patients with HBV or HCV infection. For studies that reported adjusted RR data in various multivariate-analysis models, the one with the most adequately adjusted variables were used for the meta-analysis. Data of RRs and their corresponding stand errors were calculated from 95% CIs or *p* values and logarithmically transformed to stabilize variance and normalized the distribution (27). The Cochrane's Q test was used to evaluate the heterogeneity among the included cohort studies and the estimation of the I^2^ statistic (29). Significant heterogeneity was considered if I^2^ > 50%. We used a random-effect model to synthesize the RR data because this model could incorporate the heterogeneity among the included studies and retrieve a more generalized outcome (27). For the primary outcome of HCC incidence, sensitivity analyses, by omitting one individual study at a time, were performed to test the robustness of the results (30). Predefined subgroup analyses were performed to evaluate the influences of study characteristics on the outcome, such as the viral type, age, sex, diabetic status, and cirrhotic status of the patients, the exposure time of aspirin, follow-up durations, and the adjustment of antiviral treatment and statin use. Medians of the continuous variables were used as cutoff values for defining the subgroups. The potential publication bias was assessed by visual inspection for the symmetry of the funnel plots. Moreover, Egger's regression asymmetry test (31) was also performed if at least 10 datasets were included in the meta-analysis. If a significant publication was suggested, a trim-and-fill analysis was performed. This method incorporated hypothesized unpublished studies with negative results to generate symmetrical forest plots (27). A *p*-value < 0.05 was considered statistically significant. We used RevMan (Version 5.1; Cochrane Collaboration, Oxford, UK) and STATA software for the meta-analysis and statistics.

## Results

### Literature Search

**Figure 1:**
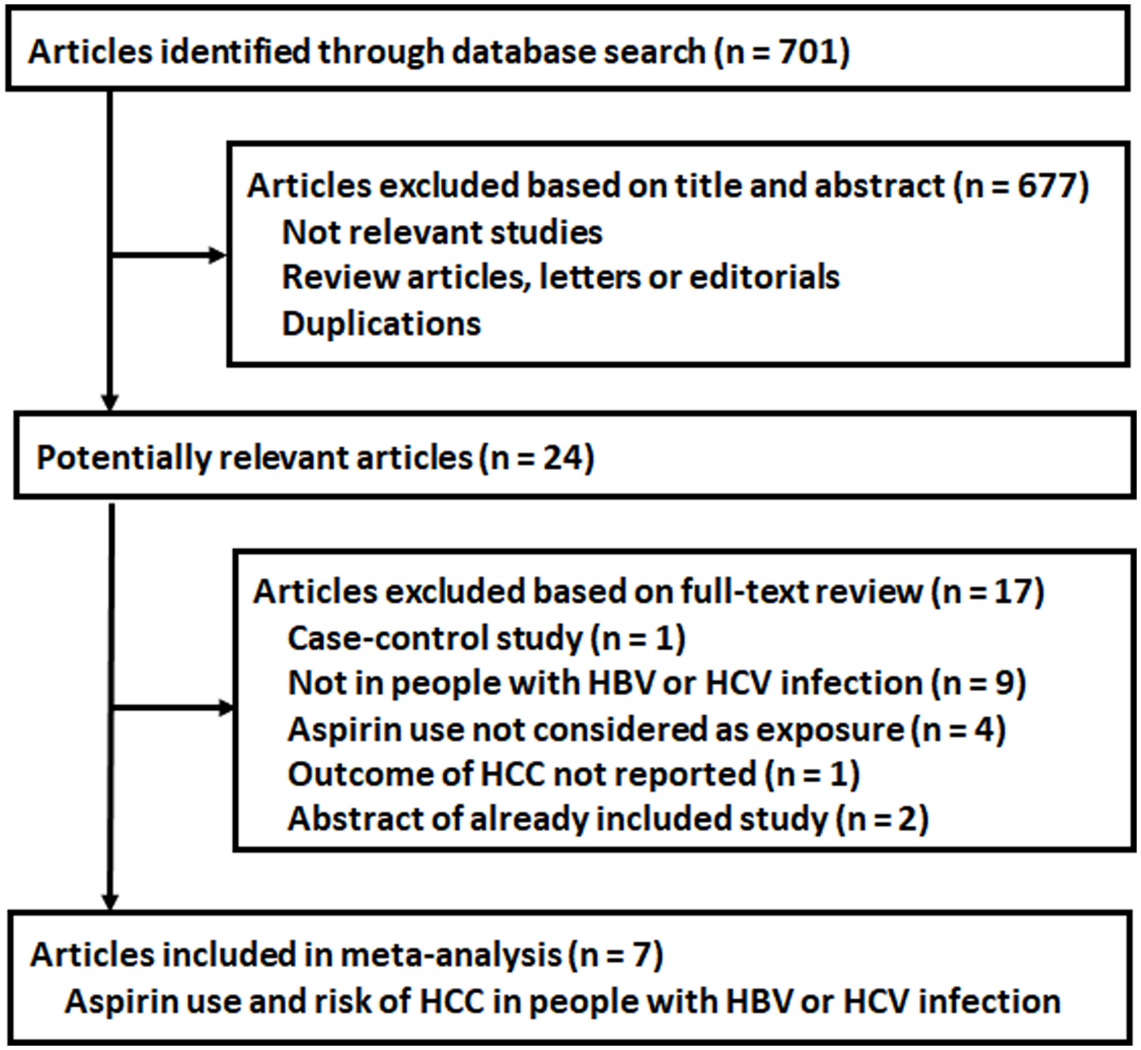
Flowchart of database search and study identification.

The process of database search is summarized in Figure 1. Briefly, 701 articles were found via initial literature search of the PubMed, Cochrane's Library, and Embase databases, and 677 were excluded through screening of the titles and abstracts mainly because they were not relevant to the purpose of the meta-analysis. Subsequently, 24 potential relevant records underwent full-text review. Of these, 17 were further excluded based on the reasons listed in Figure 1. Finally, seven articles were included (18–24).

### Study Characteristics and Quality Evaluation

The characteristics of the included studies are summarized in Table 1. All of the included studies were retrospective cohort studies except for one study, which was a prospective cohort study (24). These studies were published between 2017 and 2020 and included adult patients with HBV or HCV from South Korea (18, 20), China (19, 21–23), and Sweden (24). Because one study reported the association between aspirin use and HCC risk in patients with HBV and HCV infection separately (19), these datasets were included independently. Overall, eight datasets from seven cohort studies, with 120,945 adult patients with HBV or HCV infection, were included (18–24). The mean age of the included patients varied from 42 to 65 years, with proportions of males ranging from 44 to 73%. Aspirin use was generally validated by medicine prescription records, which were prescribed at a dose of 100 mg/day in four studies (18, 21–23), of 75 or 160 mg/day in one study (24), whereas the other two studies did not specify the dose of aspirin (19, 20). The follow-up duration varied from 4.0 to 7.9 years. Diagnosis of HCC was based on clinical data in two studies (18, 19) and the International Classification of Diseases version 9 or 10 codes in the other five (20–24). A total of 4,783 HCC cases occurred during follow-up. Potential confounding factors, such as age, sex, diabetic status, alcohol intake, comorbidities, and concurrent medications, were adjusted when presenting the outcome in all of the included studies. The Newcastle–Ottawa Scale scores of the included studies ranged from six to nine, indicating generally good study quality (Table 2).

**Table 1:** Characteristics of the included cohort studies.

**Study**	**Country**	**Design**	**Patient characteristics**	**Sample size**	**Mean age**	**Male**	**Aspirin user**	**Cirrhosis**	**Aspirin dose and validation**	**Follow-up timespan and mean durations**	**HCC validation**	**HCC cases**	**Variables adjusted**	**NOS**
					years	%		%		years				
Lee et al. (18)	South Korea	RC	Patients with HBV infection on antiviral treatment	1,674	51.9	63.0	343	12.2	Aspirin 100 mg/day for primary and secondary prevention of CVD validated by medical records	2002–2015, mean: 4.8	Serum AFP, ultrasonography, contrast-enhanced liver CT or MRI	63	Age, sex, DM, cirrhosis, Child-Pugh score, MELD score, HBeAg, ALT, albumin, total bilirubin, SCr, PT, and platelet count	8
Ho et al. (19)-HBV	China	RC	HBV patients with hypertension	7,724	57.4	66.2	3,575	78.9	Aspirin used for antiplatelet therapy	2005–2014, mean: 4	Histological confirmation or typical imaging presentation	552	Age, sex, low economic income, other comorbidities, and concurrent use of ACEI/ARB, metformin, and statin	7
Ho et al. (19)-HCV	China	RC	HCV patients with hypertension	7,873	59.5	49.3	3,349	88	Aspirin used for antiplatelet therapy	2005–2014, mean: 4.6	Histological confirmation or typical imaging presentation	503	Age, sex, low economic income, other comorbidities, and concurrent use of ACEI/ARB, metformin, and statin	7
Hwang et al. (20)	South Korea	RC	Patients with HBV or HCV infection	31,528	50.0	53.6	NR	NR	Regular aspirin prescription records (>30 days) within a 5-year before the index date	2007–2013, mean: 6.4	ICD-10	773	Age, sex, BMI, health behaviors, concurrent medication, BP, FPG,TC, socioeconomic status, and CCI	7
Lee et al. (21)	China	RC	Patients with chronic HBV infection	10,615	58.8	72.4	2,123	17.1	Regular aspirin prescription records (>90 days); Mostly (98%) of aspirin (100 mg/day) for CVD prevention	1997–2012, mean: 5	ICD-9	697	Age, sex, liver cirrhosis, DM, hyperlipidemia, hypertension, statin use, metformin use, and antiviral therapy	7
Liao et al. (23)	China	RC	Patients with newly diagnosed HCV infection	3,822	64.5	47.1	1,911	NR	Aspirin 100 mg/day for antiplatelet therapy	2000–2012, mean: 4	ICD-9	147	Age, sex, hypertension, DM, moderate or severe liver disease, MI, CHF, ischemic stroke, anti-hypertension agents, hypoglycemic agents, and heparin, other antithrombotic agents and NSAIDs	6
Lee et al. (22)	China	RC	Patients with chronic HCV infection	7,434	63.2	44.3	2,478	15.7	Regular aspirin use of 100 mg/day prescribed for >90 days	1997–2011, mean: 5	ICD-9	436	Age, sex, cirrhosis, liver decompensation, hyperlipidemia, statin use, and interferon therapy	7
Simon et al. (24)	Sweden	PC	Patients with chronic HBV or HCV infection	50,275	42.7	66.2	14,205	14.0	Regular aspirin use of 75 or 160 mg/day prescribed for >90 days	2005–2013, mean: 7.9	ICD-9	1612	Age, sex, liver disease severity, antiviral treatment, DM, hypertension, obesity, or alcohol abuse or misuse, and use of insulin, metformin, and statins	9

*HCC, hepatocellular carcinoma; NOS, Newcastle–Ottawa Scale; RC, retrospective cohort; PC, prospective cohort; HBV, hepatitis B virus; HCV, hepatitis C virus; NR, not reported; CVD, cardiovascular diseases; AFP, alpha fetoprotein; CT, computed tomography; MRI, magnetic resonance imaging; DM, diabetes mellitus; MELD, model for end-stage liver disease; ALT, alanine transaminase; SCr, serum creatinine; PT, prothrombin time; ACEI, angiotensin converting enzyme inhibitor; ARB, angiotensin II receptor blocker; BMI, body mass index; FPG, fasting plasma glucose; TC, total cholesterol; CI, Charlson comorbidity index; MI, myocardial infarction; CHF, congestive heart failure; NSAIDs, nonsteroidal anti-inflammatory drugs; ICD, International Classification of Diseases*.

**Table 2:** Quality evaluation of the included cohort studies via the NOS.

**References**	**Representativeness of the exposed cohort**	**Selection of the non-exposed cohort**	**Ascertainment of exposure**	**Outcome not present at baseline**	**Control for age and sex**	**Control for other confounding factors**	**Assessment of outcome**	**Enough long follow-up duration**	**Adequacy of follow-up of cohorts**	**Total**
Lee et al. (18)	1	1	1	1	1	0	1	1	1	8
Ho et al. (19)-HBV	0	1	1	1	1	0	1	1	1	7
Ho et al. (19)-HCV	0	1	1	1	1	0	1	1	1	7
Hwang et al. (20)	1	1	0	1	1	1	0	1	1	7
Lee et al. (21)	1	1	1	1	1	0	0	1	1	7
Liao et al. (23)	1	1	0	1	1	0	0	1	1	6
Lee et al. (22)	1	1	1	1	1	0	0	1	1	7
Simonet et al. (24)	1	1	1	1	1	1	1	1	1	9

*NOS, Newcastle–Ottawa Scale; HBV, hepatitis B virus; HCV, hepatitis C virus*.

### Primary Outcome

**Figure 2:**
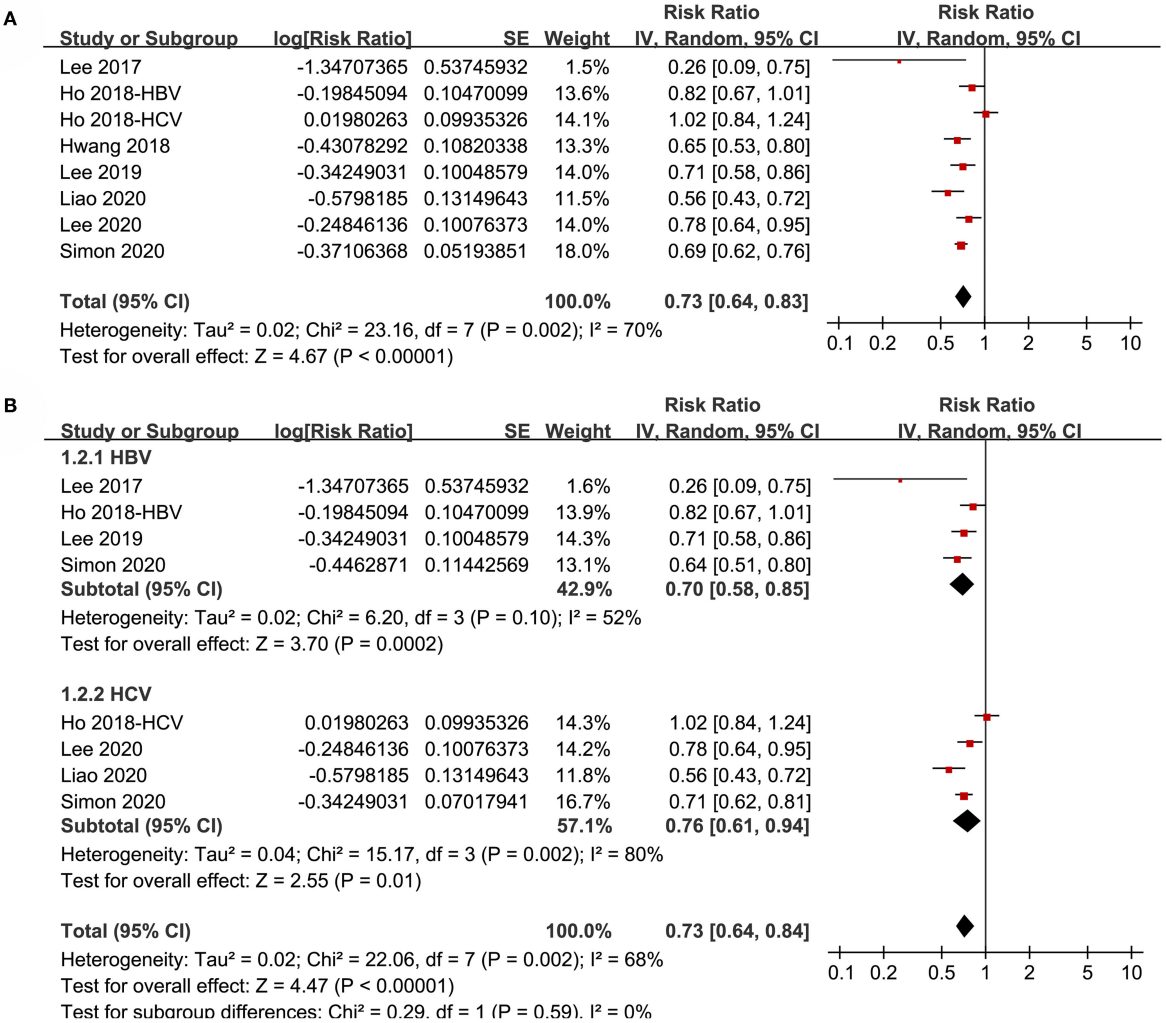
Forest plots for the meta-analysis of the association between aspirin use and HCC incidence in patients with HBV or HCV infection: **(A)** overall meta-analysis. **(B)** Subgroup analyses according to the viral type.

Pooled results of eight datasets (18–24) with a random-effect model showed that aspirin use was associated with a reduced risk of HCC in patients with HBV or HCV infection (RR: 0.73, 95% CI: 0.64 to 0.83, *p* < 0.001; Figure 2A) with significant heterogeneity (p for Cochrane's Q test = 0.002, I^2^ = 70%). Results of sensitivity analyses by omitting one dataset at a time did not significantly change the results (RR: 0.70 to 0.75, *p* all < 0.05). Specifically, excluding the only prospective cohort study showed similar results (RR: 0.73, 95% CI: 0.62 to 0.87, *p* < 0.001; I^2^ = 72%).

**Figure 3:**
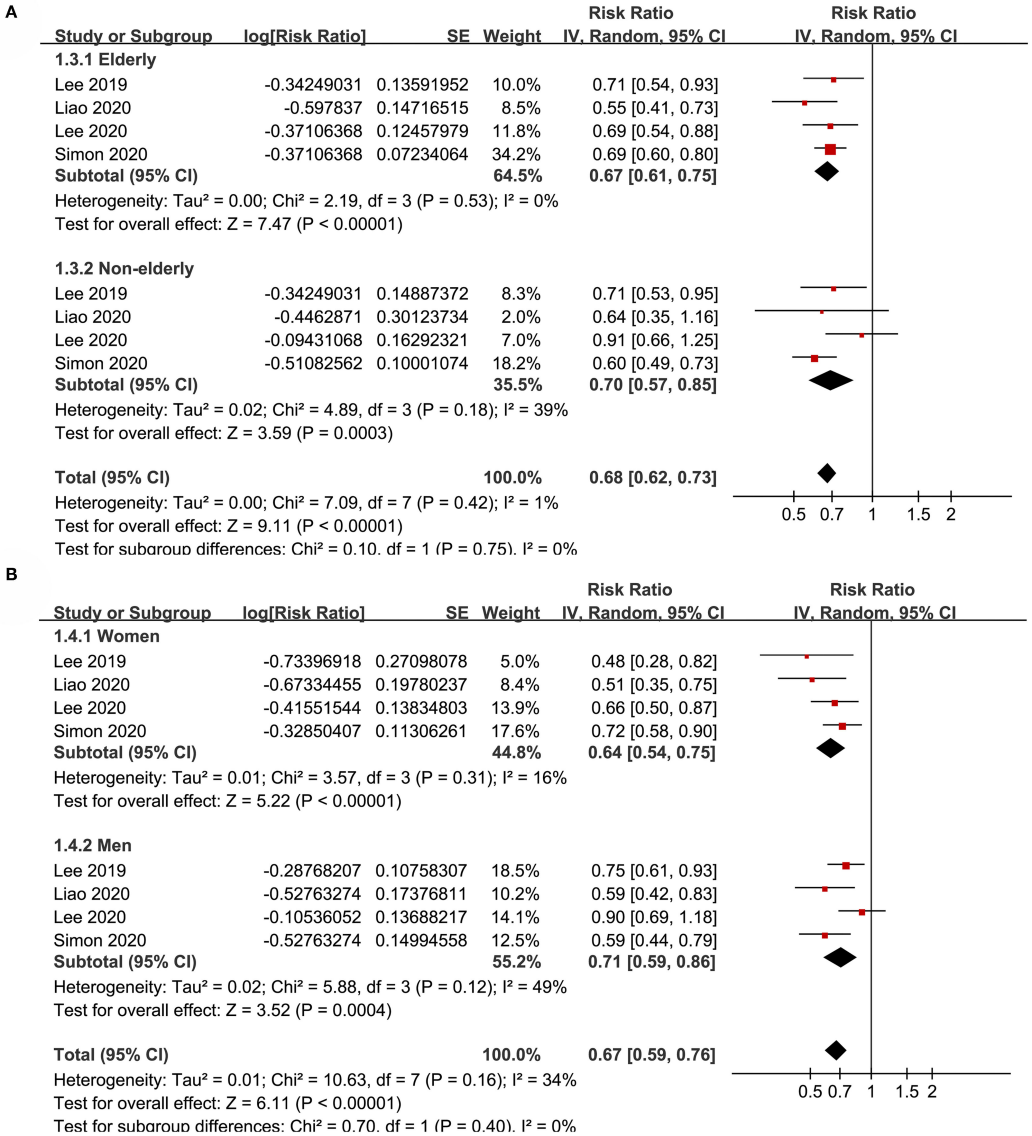
Subgroup analyses for the meta-analysis of the association between aspirin use and HCC incidence in patients with HBV or HCV infection. **(A)** Subgroup analyses according to the age of the patients. **(B)** Subgroup analyses according to the gender of the patients.

**Figure 4:**
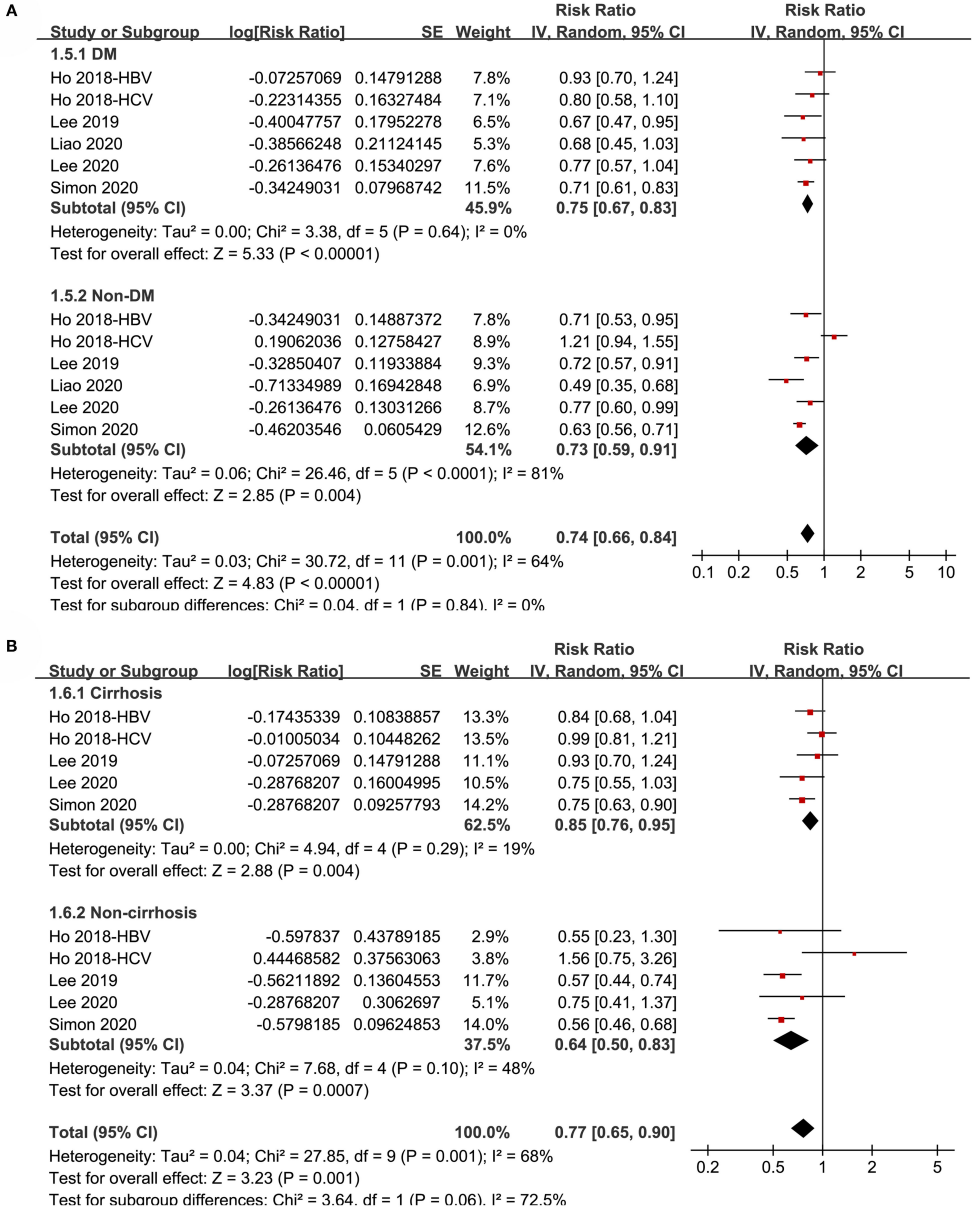
Subgroup analyses for the meta-analysis of the association between aspirin use and HCC incidence in patients with HBV or HCV infection. **(A)** Subgroup analyses according to diabetic status. **(B)** Subgroup analyses in patients with and without cirrhosis.

**Figure 5:**
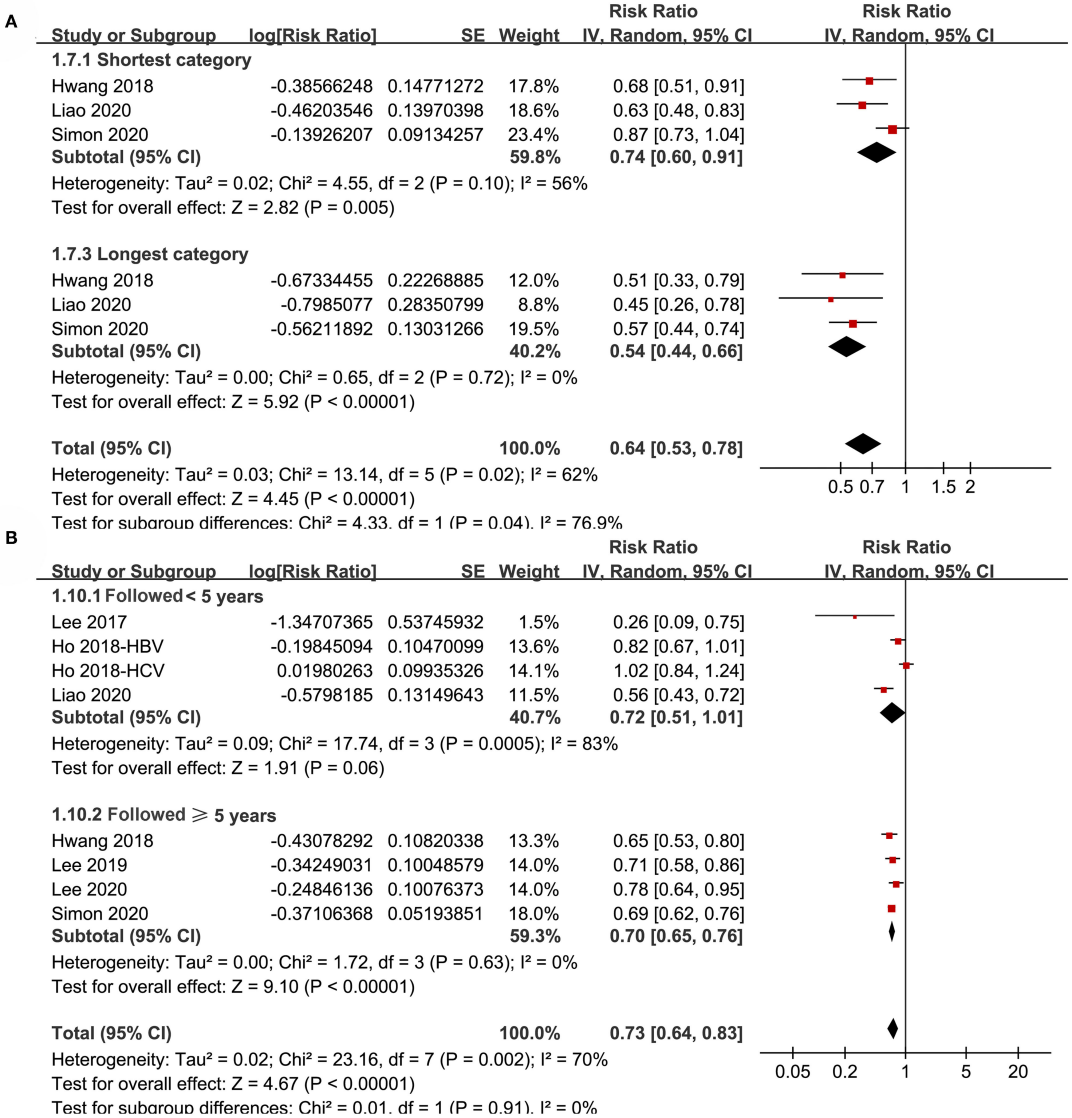
Subgroup analyses for the meta-analysis of the association between aspirin use and HCC incidence in patients with HBV or HCV infection. **(A)** Subgroup analyses according to the exposure duration of aspirin. **(B)** Subgroup analyses according to the follow-up duration.

**Figure 6:**
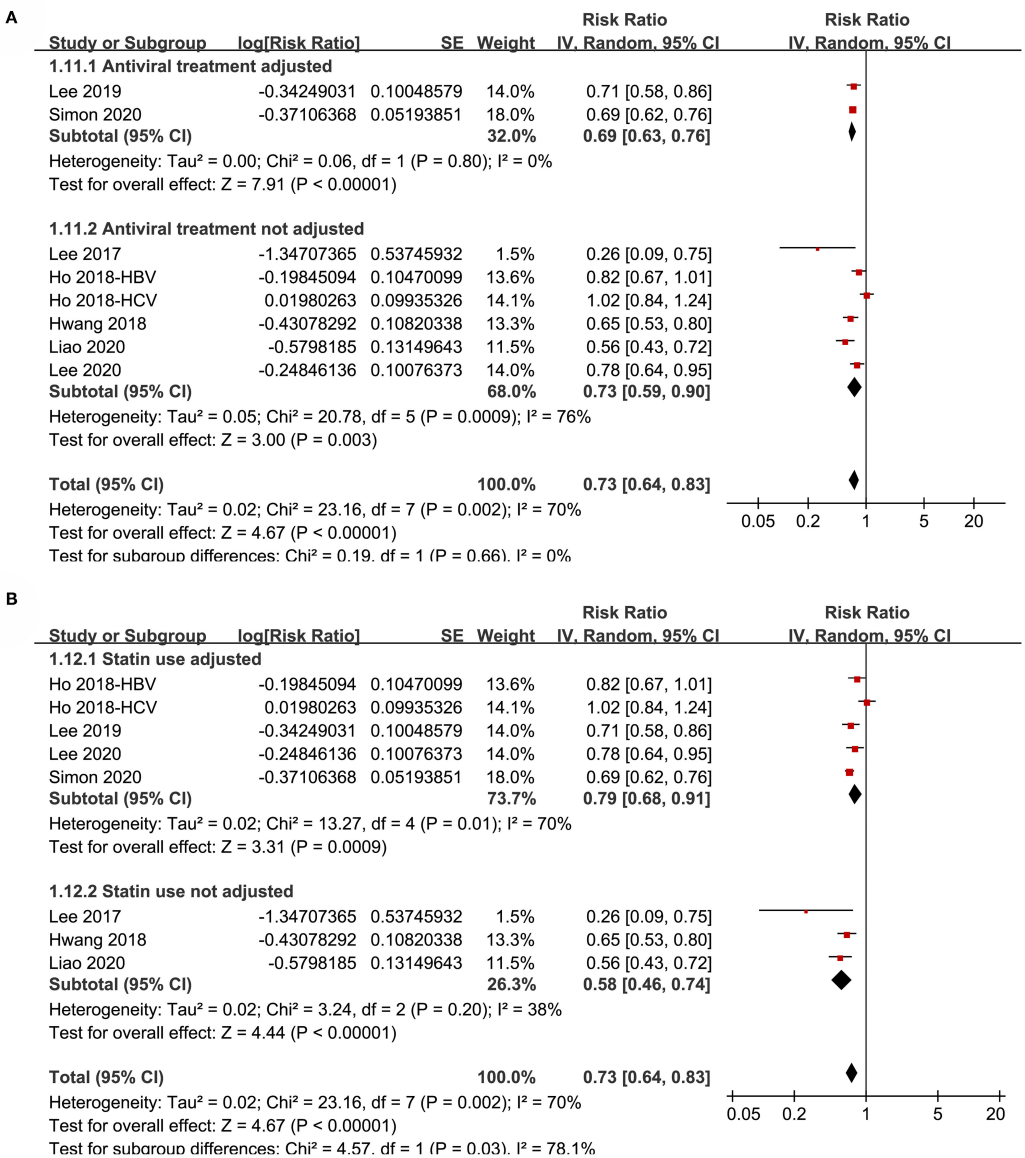
Subgroup analyses for the meta-analysis of the association between aspirin use and HCC incidence in patients with HBV or HCV infection. **(A)** Subgroup analyses according to the adjustment of antiviral treatment. **(B)** Subgroup analyses according to the adjustment of statin use.

Subgroup analyses showed that aspirin use was associated with a reduced HCC incidence in patients with HBV (RR: 0.70, 95% CI: 0.58 to 0.85, *p* < 0.001; I^2^ = 52%) and HCV infection (RR: 0.76, 95% CI: 0.61 to 0.94, *p* = 0.01; I^2^ = 80%; p for subgroup difference = 0.59; Figure 2B). Definitions for the elderly were in accordance with the criteria of the original studies, which were defined as >60 years in two studies (21, 23) and >65 in the other two studies (22, 24). Results of subgroup analyses also showed that aspirin use was associated with a reduced HCC risk in patients with HBV or HCV infection regardless of the age, sex, diabetic status, and cirrhotic status of the patients (Figures 3A,B, 4A,B). Because different cutoff values for the aspirin exposure time were used in the original studies, we compared the association between aspirin use and HCC risk in the infected patients with the longest and the shortest categories of aspirin exposure time. Results showed that the association was stronger in patients with the longest categories of aspirin exposure time (RR: 0.54, 95% CI: 0.44 to 0.66, *p* < 0.001; I^2^ = 0%) compared with that in patients with the shortest categories (RR: 0.74, 95% CI: 0.60 to 0.91, *p* = 0.005; I^2^ = 56%; *p* for subgroup difference = 0.04; Figure 5A). Moreover, subgroup analyses according to the follow-up durations, adjustment of antiviral treatment, and adjustment of statin use consistently showed a significant association between aspirin use and reduced risk of HCC in these patients (Figures 5B, 6A,B). However, the association between aspirin use and reduced risk of HCC was weakened in studies after adjustment of statin use (RR: 0.79, 95% CI: 0.68 to 0.91, *p* < 0.001; I^2^ = 70%) compared with that in studies without adjustment of statin use (RR: 0.58, 95% CI: 0.46 to 0.74, *p* < 0.001; I^2^ = 38%; p for subgroup difference = 0.03; Figure 6B).

### Secondary Outcome

**Figure 7:**
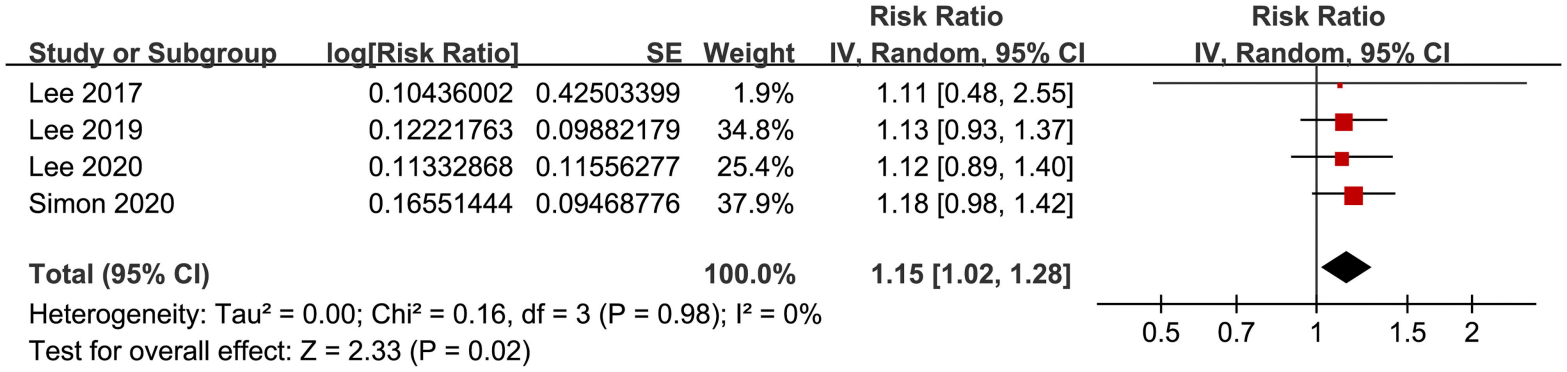
Forest plots for the meta-analysis of the association between aspirin use and GIB incidence in patients with HBV or HCV infection.

The meta-analysis by pooling the results of four studies (18, 21, 22, 24) showed that aspirin use was associated with an increased risk of GIB in patients with HBV or HCV infection (RR: 1.15, 95% CI: 1.02 to 1.28, *p* = 0.02; I^2^ = 0%; Figure 7). No subgroup analysis was performed because only four studies were included.

### Publication Bias

**Figure 8:**
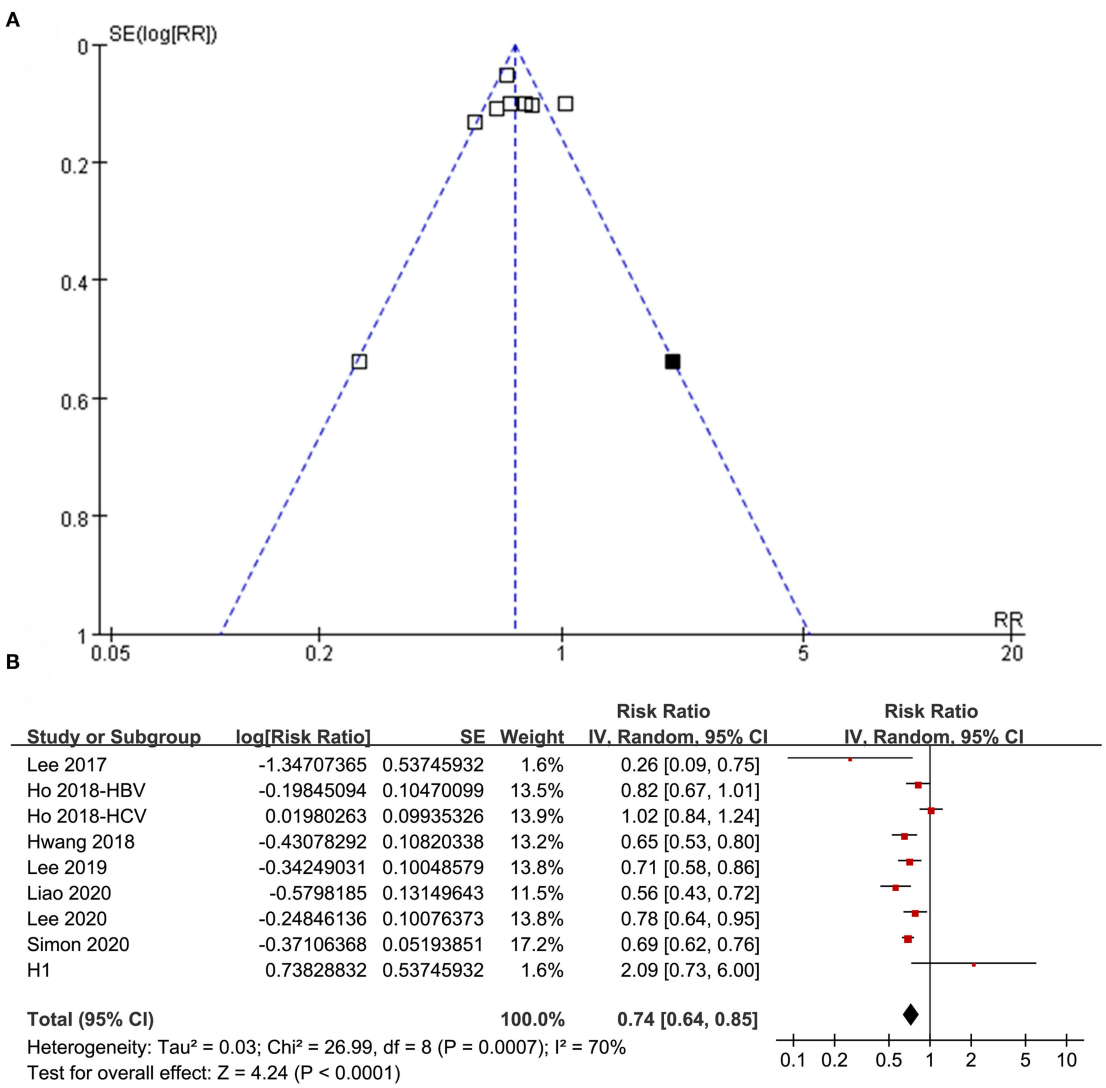
Publication bias analysis. **(A)** Funnel plots with trim-and-fill analysis for the association between aspirin use and HCC incidence in patients with HBV or HCV infection. **(B)** Forest plots incorporating the hypothesized study for the meta-analysis of the association between aspirin use and HCC incidence in patients with HBV or HCV infection. Black square in **(A)** represents the hypothesized unpublished study with negative results, and incorporating this study achieved symmetrical funnel plots. Study H1 in **(B)** represents the datasets of the hypothesized unpublished study and including this study in the meta-analysis did not change the results significantly.

The funnel plots regarding the association between aspirin use and HCC risk in patients with HBV or HCV infection are shown in Figure 8A. The funnel plots were asymmetrical on visual inspection, suggesting the potential risk of publication bias. Egger's regression test was not performed because only eight datasets were included. With a trim-and-fill analysis, one hypothesized study with negative results was imputed (Figure 8A) to achieve funnel plots. Including this study into the meta-analysis did not significantly change the results (RR: 0.74, 95% CI: 0.64 to 0.85, *p* < 0.001; I^2^ = 70%; Figure 8B). Publication bias regarding the association between aspirin use and GIB risk was unable to be analyzed due to the limited studies included.

## Discussion

In this meta-analysis cohort study, we found that aspirin use was independently associated with a reduced risk of HCC in patients with HBV or HCV infection. Subgroup analyses showed that the significance of the association was not affected by the viral type, age, sex, diabetic status, the cirrhotic status of the patients, and follow-up durations, suggesting the robustness of the findings. Although consistent results were obtained in subgroup analyses according to the adjustment of antiviral treatment and statin use, the association between aspirin use and reduced risk of HCC in these patients was weakened in studies with adjustment of statin use compared with that in studies without adjustment of statin use. In addition, aspirin use was also associated with an increased risk of GIB in these patients. Taken together, these findings indicated that aspirin use was independently associated with a reduced risk of HCC in patients with HBV or HCV infection, whereas the risk of GIB may be increased. Randomized controlled trials (RCTs) should be performed to validate these findings and weigh the benefit and risk of aspirin use in patients with HBV or HCV infection.

As far as we know, our study is the first meta-analysis that focused on the association between aspirin use and HCC risk in patients with HBV or HCV infection. We only included cohort studies with multivariate analyses, which therefore provided an independent association between aspirin use and a reduced risk of HCC in these patients. Because no RCTs have been performed regarding the preventative efficacy of aspirin for HCC, even in high-risk patients such as those with HBV or HCV infection, our study highlights the necessity to perform such studies. Sensitivity analyses and multiple subgroup analyses were performed to evaluate the robustness of the results. We found that the results of the meta-analysis were not significantly affected by either of the individual included studies. Besides, the association between aspirin use and reduced HCC risk was significant regardless of the age, sex, viral status, diabetic status, the cirrhotic status of the patients, and follow-up durations. Moreover, the association between aspirin use and reduced HCC risk was not significantly affected by the adjustment of antiviral treatment, whereas significantly weakened in studies with adjustment of statin use compared with that in studies without adjustment of statin use. A previous meta-analysis showed that statin use was associated with a reduced incidence of HCC in patients with HBV/HCV infection. Moreover, aspirin is commonly prescribed in conjunction with a hypocholesterolemic agent such as a statin, which may confound the association between aspirin and reduced HCC risk in these patients. Our subgroup analyses showed that the association between aspirin and reduced HCC risk remains significant in subgroups, including studies with adjustment of statin use, which further supported a potential independent association between aspirin and reduced HCC risk in these patients.

The mechanisms underlying the association between aspirin use and reduced HCC risk may be multifactorial. An early study showed that aspirin inhibited HCV RNA and protein expression through cyclooxygenase-2 signal pathways (32), contributing to their anticancer efficacy in HCV-related HCC. Besides, aspirin could inhibit HCV entry via downregulation of claudin-1, thereby further inhibiting HCV replication, which could also be responsible for a reduced HCC risk in patients with HCV infection (33). Another study in the mice model of chronic HBV infection showed that antiplatelet therapy with aspirin or clopidogrel prevented HCC and improved survival in mice model of chronic HBV infection via amelioration of immune-mediated chronic liver injury by overactivated platelets (34, 35). Moreover, cirrhosis has been associated with an increased risk of HCC in patients with HBV or HCV infection (36). Aspirin use has been related to reduced liver fibrosis progression (37), which may be another important mechanism underlying its association with reduced HCC risk. The exact mechanisms underlying the association between aspirin use and reduced HCC risk in patients with HBV or HCV infection deserve further studies.

We found that aspirin use may be associated with an increased risk of GIB in patients with HBV or HCV infection. These results should be validated because only four datasets were available. Currently, we could not determine the potential patient characteristics and concurrent medication on the association between aspirin use and GIB risk, as these data were rarely reported in the included studies. For example, decompensated cirrhotic patients are vulnerable to variceal bleeding, and the use of aspirin may expose these patients to a higher risk of GIB (38). Therefore, it could be hypothesized that aspirin may increase the risk of GIB primarily in HBV- or HCV-infected patients with cirrhosis. Accordingly, prophylactic medications for GIB, such as proton pump inhibitors (PPIs), are often prescribed in these patients, particularly when aspirin is indicated. However, the use of PPIs in cirrhotic patients has been related to increased risks of adverse events such as hepatic encephalopathy, spontaneous bacterial peritonitis, and possibly HCC and mortality (39, 40). Therefore, the influence of aspirin use in cirrhotic patients with HBV or HCV infection may be much more complicated in real-world clinical practice, which warrants delicately designed RCTs to evaluate the potential benefits on HCC and risks of GIB.

Our study has limitations that should be noticed when interpreting the results. Firstly, significant heterogeneity was detected among the included studies. Although some subgroup analyses were performed to evaluate the patient characteristics on the outcome, we could not exclude other factors that may contribute to the heterogeneity, such as concurrent medications, alcohol intake, dietary factors, among others (41). Secondly, limited studies were included for the subgroup analyses, the results of which should be interpreted cautiously. Thirdly, antiviral therapy outcome was not considered when subgroup analysis according to the adjustment of antiviral therapy was performed. This is because none of the included studies provided any data according to the outcome of antiviral therapy. Fourthly, publication bias may exist, although a trim-and-fill study was performed and showed similar results. Furthermore, although we included studies with adjusted data, three are chances that residual factors may confound the association. Besides, a causative association between aspirin use and reduced HCC risk in HBV- or HCV-infected patients could not be derived based on our study, as this is a meta-analysis of observational studies. In addition, a potential time-dependent and dose-dependent association between aspirin use and reduced HCC in HBV- or HCV-infected patients was not observed due to the inconsistent cutoffs for the duration and dose of aspirin exposure in the included studies. Moreover, the loss of follow-up in the cohort study is an important issue that affects the outcome, which may also affect the results of our meta-analysis. Finally, the net benefits or harms of aspirin use in HBV- or HCV-infected patients could not be determined based on our study. Large-scale RCTs should be performed to weigh the benefit and risk of aspirin use in patients with HBV or HCV infection considering the cirrhotic status and concurrent medications, such as PPIs.

In conclusion, the results of the meta-analysis showed that aspirin use was independently associated with a reduced risk of HCC in patients with HBV or HCV infection, whereas the risk of GIB may be increased. Large-scale RCTs are needed to validate these findings and weigh the benefit and risk of aspirin use in patients with HBV or HCV infection.

## Data Availability Statement

The datasets presented in this study can be found in online repositories. The names of the repository/repositories and accession number(s) can be found in the article/supplementary material.

## Author Contributions

XL conceived and designed the study and drafted the paper. XL and SW selected the studies and collected the data. XL and YY analyzed data. All authors interpreted the results, revised the draft paper and read and approved the final version of the manuscript.

## Conflict of Interest

The authors declare that the research was conducted in the absence of any commercial or financial relationships that could be construed as a potential conflict of interest.
